# Plasticity enhancement in pharmaceutical drugs by water of crystallization: unusual slip planes

**DOI:** 10.1107/S205225251900890X

**Published:** 2019-06-28

**Authors:** C. Malla Reddy

**Affiliations:** a Department of Chemical Sciences, Indian Institute of Science Education and Research (IISER), Kolkata, Mohanpur Campus, Mohanpur, Nadia-741246, West Bengal, India

**Keywords:** plastic bending, hydrates, single-crystal transformations

## Abstract

Khandavilli *et al.* [(2019), *IUCrJ*, **6**, 630–634] show the superior plasticity in hydrates of the pharmaceutical drugs, pregabalin and gabapetin, compared with their anhydrous forms. The water in the structure is believed to act as a lubricating agent in the packing of hydrates, thus facilitating slippage of molecules in the plastic bending of the crystals under external mechanical stress.

Metals, metal-based alloys, ceramics and polymeric materials have been known to show exceptional plasticity (or permanent deformation) for many decades. Such plasticity phenomena have also been studied in molecular materials, such as globular hydro­carbons with predominantly dispersive interactions, where the plastic crystal is a mesophase between the solid and liquid states (Sherwood, 1979 ▸). In the mid-19th century, solid-state chemists also studied the plasticity in organic crystals in an attempt to draw parallels with their inorganic counterparts. However, technological limitations in determining molecular-level structural modifications, as well as molecular diversity associated with varying shapes, sizes and non-covalent interactions, make it nearly impossible to extrapolate the simplistic models drawn purely from near-spherical atomic solids with predominantly high symmetry and near isotropic interactions. Hence, any attempts to address modern-day practical problems, such as those for pharmaceuticals and fine chemicals, quickly fail in the absence of structure–mechanical property correlation in molecular crystals (Reddy *et al.*, 2010 ▸; Sun, 2009 ▸; Mishra *et al.*, 2016 ▸; Saha *et al.*, 2018 ▸). For example, it remains hard to predict many problems that arise during mechanical actions in various production processes of pharmaceutical drugs. Also, organic electrical and optical materials with an ability to be cast into any desired shape would be of paramount importance to modern flexible devices, but it is genuinely challenging to design such mechanically adaptable functional solids from general rules of deformation and known dislocation mechanisms in inorganic materials. Hence, recent attempts to investigate structure–mechanical property correlations have attracted the attention of the crystal engineering community, working in solid-state pharmaceutical chemistry and optoelectronic materials. Plasticity and elasticity of materials immensely influence various bulk processes in drug product development, such as milling, mechanical stability, flowability, granulation, tablet manufacturing and so on (Ghosh & Reddy, 2012 ▸; Saha *et al.*, 2018 ▸; Sun, 2009 ▸). For instance, the lack of plasticity of high-dosage drugs, where the concentration of the drug dominates the composition of a tablet, is a concern.

The terms elasticity, plasticity and brittleness in molecular solids typically depend on their intrinsic molecular arrangements and the rate of applied load (Reddy *et al.*, 2005 ▸). Thereby, the crystals may bend on one or two pairs of crystal faces, twist or shear. Crystal morphology also plays an important role in mechanical deformation, but internal structure remains the dominant factor.

Plasticity in molecular crystals can generally be obtained when the crystals are anisotropic and have facile slip planes, which weakly interact through dispersive noncovalent interactions, involving functional groups such as methyl, *tert*-butyl, meth­oxy, chloro, bromo and aromatic groups among many others (Reddy *et al.*, 2006 ▸; Mukherjee & Desiraju, 2014 ▸; Panda *et al.*, 2015 ▸; Naumov *et al.*, 2015 ▸; Krishna *et al.*, 2016 ▸). The dispersive nature of these weak interactions and low rugosity of the slip layer along the slip direction play a substantial role during plastic bending (Krishna *et al.*, 2015 ▸). The weak interaction planes with high structural rugosity lead to high mechanical resistance (Bryant *et al.*, 2018 ▸). Rough potential energy surfaces of the weak interaction planes may still lead to energetic interlocking. Recently, the quasi-isotropic crystal packing and interaction topology in di­methyl sulfone crystals have also been associated with exceptional plasticity (Thomas *et al.*, 2017 ▸). In contrast, crystals with rigid supramolecular architectures undergo brittle fracture. When subjected to a shear stress exceeding the fracture stress, the crystals would fracture instead of deforming plastically or elastically. The presence of dispersive intermolecular interactions seems to be an important factor for high elasticity in molecular crystals, which act as structural buffers during bending (Ghosh & Reddy, 2012 ▸; Saha *et al.*, 2018 ▸; Takamizawa & Miyamoto, 2014 ▸). However, since crystal arrangements have been found to be diverse in reported elastic crystals, the general structural models for elastic crystals are yet to be established.

Reporting in **IUCrJ**, Khandavilli *et al.* (2019 ▸) have shown a rare plastic bending behaviour in hydrate forms of two anticonvulsant zwitterionic drugs, pregabalin and gabapentin. Interestingly, the anhydrous forms of both drugs, despite close structural similarity, do not bend plastically. The zwitterions are intercalated by water molecules to form two-dimensional layers which stack on top of each other. The anhydrous forms of the respective drugs are brittle, corresponding to interdigitating structures that do not allow plasticity. In contrast, the plastic bending in hydrates is believed to be facilitated by the reorganization of noncovalent bonds formed by water molecules. This study is in contrast to the weak interaction-based slip-plane models found commonly in other plastically bent crystals. The presence of water molecules in the crystal structure plays a crucial role in the plasticity of these crystals. The role of water molecules in achieving high tabletability was earlier demonstrated by Sun & Grant (2004 ▸), where water molecules sitting between molecular layers act as ‘molecular lubricants’. Other examples (Panda *et al.*, 2017 ▸) showed that the inclusion of weakly bonded water molecules in the crystal structure of carbohydrates facilitates slip during plastic deformation. Recently, a few reports have been published on the role of water molecules in obtaining twistable two-dimensional plastic crystals (Saha *et al.*, 2018 ▸; Hu *et al.*, 2019 ▸). The report by Khandavilli *et al.* (2019 ▸) contrasts with this work as the interactions involved in the case of water are strong compared with the weak interactions found in other known plastic crystals. Hence, this work raises some interesting questions related to the dynamics of water molecules and their hydrogen bonds in crystal structures (Panda *et al.*, 2017 ▸). Although hydrogen bonds involving water are much stronger, their dynamic behaviour suggests that the more important aspect in dynamics is the barrier to achieve molecular movements and not the absolute total interaction energies that hold the two layers together (Chang & Sun, 2017 ▸). As rightly noted by the authors, further studies are required to fully establish the role of water molecules in the plasticity of these crystals.

Mechanical properties are fundamental properties, much like density, optical, thermal and electrical properties. Therefore, mechanical properties are relevant to all types of materials and their importance cannot be ignored when addressing problems in practical applications. Molecular level understanding is currently lacking for most mechanical processes, such as milling and flowability, that are important in designing flexible optoelectronics and manufacturing processes in pharmaceutical, fine chemical, agrochemical industries and so on. The solutions sought currently using empirical methods are tedious and expensive. Hence, further development of a scientific basis in this regard by systematic studies, such as Khandavilli *et al.* (2019 ▸), will reduce dependency on the inefficient empirical methods currently used in industry.

## Figures and Tables

**Figure 1:**
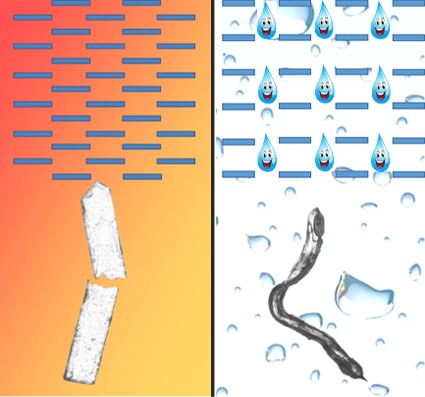
The water of crystallization in the zwitterionic drugs, pregabalin and gabapentin, facilitates high plasticity in their hydrate forms compared with the anhydrous forms, which are brittle. Reproduced from Khandavilli *et al.* (2019 ▸).
